# Investigation of a Novel Injectable Chitosan Oligosaccharide—Bovine Hydroxyapatite Hybrid Dental Biocomposite for the Purposes of Conservative Pulp Therapy

**DOI:** 10.3390/nano12213925

**Published:** 2022-11-07

**Authors:** Mingkai Cai, Jithendra Ratnayake, Peter Cathro, Maree Gould, Azam Ali

**Affiliations:** Faculty of Dentistry, Sir John Walsh Research Institute, University of Otago, P.O. Box 56, Dunedin 9054, New Zealand

**Keywords:** pulp capping, endodontic cement, biocomposite, mineral trioxide aggregate

## Abstract

This study aimed to develop injectable chitosan oligosaccharide (COS) and bovine hydroxyapatite (BHA) hybrid biocomposites, and characterise their physiochemical properties for use as a dental pulp-capping material. The COS powder was prepared from chitosan through hydrolytic reactions and then dissolved in 0.2% acetic acid to create a solution. BHA was obtained from waste bovine bone and milled to form a powder. The BHA powder was incorporated with the COS solution at different proportions to create the COS–BHA hybrid biocomposite. Zirconium oxide (ZrO_2_) powder was included in the blend as a radiopacifier. The composite was characterised to evaluate its physiochemical properties, radiopacity, setting time, solubility, and pH. Fourier-transform infrared spectroscopic analysis of the COS–BHA biocomposite shows the characteristic peaks of COS and hydroxyapatite. Compositional analysis via ICP-MS and SEM-EDX shows the predominant elements present to be the constituents of COS, BHA, and ZrO_2_. The hybrid biocomposite demonstrated an average setting time of 1 h and 10 min and a pH value of 10. The biocomposite demonstrated solubility when placed in a physiological solution. Radiographically, the set hybrid biocomposite appears to be more radiopaque than the commercial mineral trioxide aggregate (MTA). The developed COS-BHA hybrid biocomposite demonstrated good potential as a pulp-capping agent exhibiting high pH, with a greater radiopacity and reduced setting time compared to MTA. Solubility of the biocomposite may be addressed in future studies with the incorporation of a cross-linking agent. However, further in vitro and in vivo studies are necessary to evaluate its clinical feasibility.

## 1. Introduction

Pulp exposure during the management of deep caries, dental trauma, or iatrogenic damage during cavity preparation can be associated with significant morbidity in the patient. Traditionally, treatment options for a fully developed tooth with irreversible pulpitis were root canal treatment (RCT) of the affected tooth or extraction and possible prosthetic replacement. However, this can incur a significant time and financial burden on the patient [1]. Therefore, in cases of pulp exposure where the pulp is reversibly or irreversibly inflamed, conservative pulp therapy (CPT) may be utilised to avoid resorting to RCT or extraction. CPT aims to maintain the vitality of the dental pulp complex [2]. A direct pulp cap is a procedure whereby a material is placed directly over the exposed pulp to promote pulp healing and encourage reparative dentine formation. If the direct visualisation of the exposed pulp indicates that the pulp has signs of irreversible pulpitis, then pulpotomy procedures (partial removal of the pulp) can potentially maintain pulp vitality in the roots [2]. Several materials have been used as a pulp-capping agent with various levels of success in inducing the formation of reparative dentine to seal the pulp. Currently, the two most used pulp-capping materials are calcium hydroxide and mineral trioxide aggregate (MTA) [3]. However, these products are not without limitations. The main disadvantages of calcium hydroxide are its weak physical properties and gradual dissolution from the site of application [4]. MTA, as a pulp-capping material, is expensive and has a prolonged setting time of roughly 2 h and 45 min. This prolonged setting time requires the clinician to either deliver treatment in two sessions or to use a quick setting liner to protect the MTA during placement of the permanent restoration [1]. Additionally, some MTA products contain bismuth oxide as the radiopacifier, which can cause tooth staining [2].

COS is an oligomer of chitosan (β-(1→4)-linked d-glucosamine), formed as the degraded product of chitin [5,6]. Chitin, as a material, is the most abundant natural polymer, second to cellulose. It is commonly found in the exoskeletons of crustaceans and insects, and in the walls of fungi [6]. For this reason, chitin and its products can be obtained commercially at a relatively low cost, utilising waste from the seafood-processing industry [7]. Chitin as a material itself is chemically inert with a degree of acetylation over 90% and thus has limited applications [6]. Chitin is converted into chitosan via deacetylation [5,6,7]. COS can be prepared from chitosan through hydrolytic reactions that break glycosidic bonds into chitosan molecules. Compared to chitosan, COS is more readily soluble in water, has a lower viscosity, and has better absorbability, which render it more favourable for therapeutic applications [5,6]. In addition, COS is nontoxic and biodegradable [6]. Numerous recent studies described COS’s biological activities, including anti-inflammation, tissue regeneration promotion, and antimicrobial properties [5]. COS is also suitable for tissue engineering and drug/gene delivery [6]. Because of these beneficial properties, COS and its derivatives were investigated for use in biomedical, pharmaceutical, nutraceutical, and cosmeceutical applications with promising results [6]. Hydroxyapatite (HA) forms the main constituent of bones, enamel, and dentine. HA as a biomaterial is bioactive and osteoconductive [8]. For this reason, it was extensively studied in both the medical and dental fields for its potential therapeutic effects. Hydroxyapatite can be synthesised chemically or extracted from natural sources. Naturally derived HA can be extracted from biological waste products such as bovine bones, providing an economical source of HA [9,10]. BHA demonstrates a similar architecture to the HA found in human bones and teeth, with trace elements of sodium, magnesium, and fluoride [10]. Given the limitations of current pulp-capping agents, this study aimed to develop an experimental injectable composite material using chitosan oligosaccharide (COS) and bovine-derived hydroxyapatite (BHA), and characterise its physicochemical properties for use as a pulp-capping material.

## 2. Materials and Methods

### 2.1. Preparation of COS

Chitosan powder (1.5 g) was added into a 250 mL Erlenmeyer flask containing 50 mL of 2% (*v*/*v*) acetic acid. Then, 1 mL of 30% hydrogen peroxide (Sigma Aldrich, Christchurch, New Zealand) was then added to the aqueous solution of chitosan, and the solution was mixed with a metal rod to break up any large clumps. The reaction was then conducted at 70 °C for 25 min in a microwave reactor (MCR-3, KEDA Instrument (Zhengzhou) Co., Ltd., Beijing, China at 800 W microwave radiation power. Once the reaction had been completed, the solution was placed on ice to eliminate any possible further thermal effects and chemical reactions. Next, the pH of the solution was adjusted to 7 by adding 1 M NaOH (Sigma Aldrich, New Zealand) to the solution. Subsequently, a fivefold volume (250 mL) of absolute ethanol was added to the reaction mixture to obtain a white precipitate, and this solution was left for an hour to remove salts and the remaining hydrogen peroxide. Following that, the white precipitate was collected by centrifuging the solution at 4000 rpm for 15 min. This white precipitate was washed with distilled water three times, centrifuging to recollect the white precipitate after each wash. After the third wash, the white precipitate was resuspended in distilled water, and the solution was frozen using liquid nitrogen for homogeneous freezing. This was placed in a freeze dryer overnight to yield the final COS powder. The method mentioned above for COS preparation was repeated until the desired volume of COS had been produced.

### 2.2. Preparation of BHA

BHA was obtained following the protocol outlined by Ratnayake et al. [10,11,12]. In brief, cancellous bone was obtained from the condyle portion of bovine femurs and sectioned into 2 × 2 × 2 cm cubes using a bandsaw. These cubes then underwent a defatting procedure whereby they were cooked in a stainless-steel pressure cooker at 15 psi for 2 h, followed by soaking in 0.1 M NaOH solution for 12 h at 70 °C. Lastly, the cubes were heated in water for 5 min in a domestic microwave oven (2.45 GHz, 1100 W, Samsung). Once the defatting procedure was complete, the cubes underwent a deproteinisation procedure. The bone cubes were deproteinised via subcritical water extraction. The bone cubes were heated in a cylindrical hydrothermal pressure vessel at 180 °C for 1 h and sintered at 650 °C for 6 h [10,11]. The resultant processed cubes were converted into a fine powder using a ball mill.

### 2.3. Synthesis of Experimental COS-BHA Pulp-Capping Material

Different blends of COS, BHA, and zirconium oxide (Sigma Aldrich, New Zealand) (ZrO_2_) were tested, altering the proportion via the weight of the COS solution and BHA to find the ratio that yielded the most promising results as a potential injectable pulp-capping material. ZrO_2_ was added into the blend at a fixed ratio of 35% by weight for all the samples as a radiopacifier [13]. A 2% (wt) COS solution was produced by dissolving 1 g of the synthesised COS powder into 49 mL of 0.2% acetic acid solution that solubilised the COS powder better than pure distilled water did alone. This COS solution was used in the formulations to achieve a workable paste when mixed with the BHA and ZrO_2_ powders. The proportions of each investigated sample are outlined in Table 1.

The sample that yielded a homogeneous mixture and demonstrated the best handling characteristics was selected for further investigation and material characterisation.

### 2.4. Chemical Characterisation

#### Fourier Transform Infrared Spectroscopy (FTIR)

COS powder, BHA, ZrO_2_, and a sample of the experimental pulp-capping material were investigated via FTIR spectroscopy to characterise their chemical compositions. An FTIR spectrometer (ATR-FTIR Alpha II, Bruker, Auckland, New Zealand) in the range of 400–4000 cm^−1^ was used for analysis.

#### Inductively Coupled Mass Spectrometry (ICP-MS) Analysis

The analysis of the elemental composition of the experimental pulp-capping agent was performed through ICP-MS (Agilent 7900 (Santa Clara, CA, USA)). To achieve this, the sample required digesting in concentrated nitric acid (HNO_3_) (Sigma Aldrich, New Zealand), hydrofluoric acid (Hf) (Sigma Aldrich, New Zealand), and hydrochloric acid (HCl) (Sigma Aldrich, New Zealand).

#### Scanning Electron Microscopy (SEM)/Energy-Dispersive X-ray Spectroscopy (EDX) Analysis

A scanning electron microscope (SEM) coupled with X-ray analysis (EDS) (JEOL 6700 F FESEM JEOL Ltd., Tokyo, Japan) was used to obtain photomicrographs of the experimental pulp-capping biocomposite after the initial setting, and to identify the elemental composition of the sample. The sample was prepared for observation under SEM by mounting it onto an aluminium stub with double-sided carbon tape and coating the sample with roughly 10 nm of gold–palladium (Au–Pd). SEM imaging and EDX analysis were performed with field emission (Zeiss Sigma VP FEG SEM; Zeiss, Jena, Germany).

### 2.5. Physical Properties

#### Determination of pH and Solubility

pH indicator strips (MColorpHast™ Alkalit^®^ 7.5–14.0, Auckland, New Zealand) were used to measure the pH of the unset sample following its synthesis. Once the material had been set, 1.5 g of the sample was placed into 20 mL of Hank’s balanced salt solution (HBSS) with an initial pH of 7.4. The pH of the solution was then measured daily over 2 weeks using a calibrated pH probe (Hanna Instruments pH 209, Bench top pH meter (Italy)).

To assess the solubility of the sample, the structural integrity of the set material placed in HBSS was visually observed over the same 2-week period.

#### Determination of Setting Time

To measure the setting time, the material was freshly produced and then packed into a circular plastic mould spanning 10 mm in diameter and 5 mm in depth. A Vicat apparatus with a flat-ended 1.0 mm and 300 mg weight was then used to indent the sample at 10 min intervals. The setting time was defined as the time that it took for the needle to no longer cause an indentation in the material. The test was repeated three times, and the average setting time was measured.

#### Determination of Radiopacity

To assess the clinical acceptability of the experimental pulp-capping agent, the samples of the set material were radiographed alongside MTA. The samples of the unset hybrid biocomposite were placed into circular plastic moulds spanning 10 mm in diameter and 5 mm in depth, and allowed to set. MTA was also packed into these circular plastic moulds to ensure that an equal amount of each material was obtained for comparison. The set material was removed from the moulds and placed alongside MTA on a size 2 photostimulable storage phosphor (PSP) plate (Dentsply Sirona, Charlotte, NC, USA). The radiograph was captured on film at a focal distance of approximately 30 cm from a Heliodent Plus (Dentsply Sirona) X-ray source (70 kVp, 7 mA, 0.10 s). The PSP plate was then developed using a Xios PSP Scanner (Dentsply Sirona, Bensheim, Germany) and viewed using Sidexis imaging software (version 4, Dentsply Sirona, Dublin, Ireland).

## 3. Results

### 3.1. Selection of the Appropriate Experimental Pulp-Capping Material

The proportion of raw materials outlined in Sample 4 yielded a biocomposite with the best handling characteristics (Table 1). For this reason, Sample 4 was characterised to evaluate physiochemical properties, radiopacity, setting time, solubility, and pH.

### 3.2. FTIR Analysis of COS, BHA, ZrO_2_, and the Biocomposite (Sample 4)

**Figure 1:**
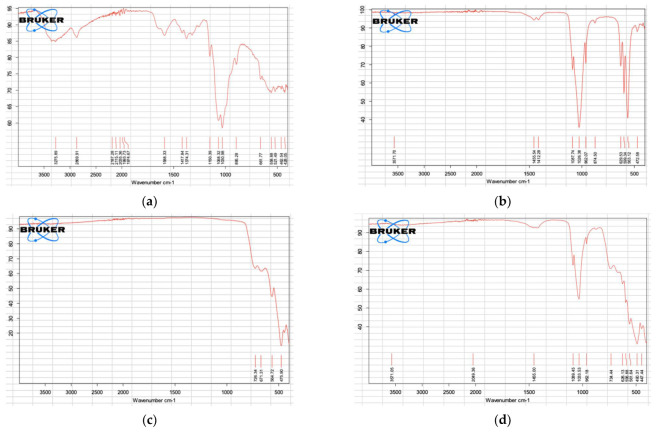
FTIR spectra. (**a**) COS. (**b**) BHA. (**c**) ZrO_2_. (**d**) Experimental pulp-capping agent COS-BHA.

The FTIR spectra for COS, BHA, ZrO_2_, and the selected experimental pulp-capping material are shown in Figure 1. The spectra for COS, BHA, and ZrO_2_ demonstrate prominent bands associated with each respective material [14,15,16]. The FTIR spectrum for the selected hybrid biocomposite demonstrates peaks overlapping the spectra of COS, BHA, and ZrO_2,_ indicating the formation of a composite of the combined raw materials.

### 3.3. ICP-MS Biocomposite Analysis

Results from the ICP-MS analysis of the experimental pulp-capping material (Sample 4) after digesting in HNO_3_, HF, and HCl are outlined in Table 2.

### 3.4. SEM-EDX Analysis

**Figure 2:**
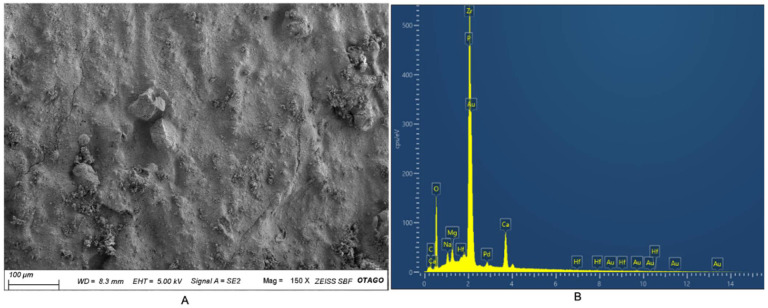
(**A**) SEM photomicrograph of the experimental pulp-capping material, scale bar = 100 μm. (**B**) EDX spectra of the hybrid biocomposite.

The SEM photomicrograph of the hybrid biocomposite and its EDX spectra are presented in Figure 2. The composition of the hybrid biocomposite as determined via SEM-EDX is outlined in Table 3.

### 3.5. pH and Solubility of Sample 4

**Figure 3:**
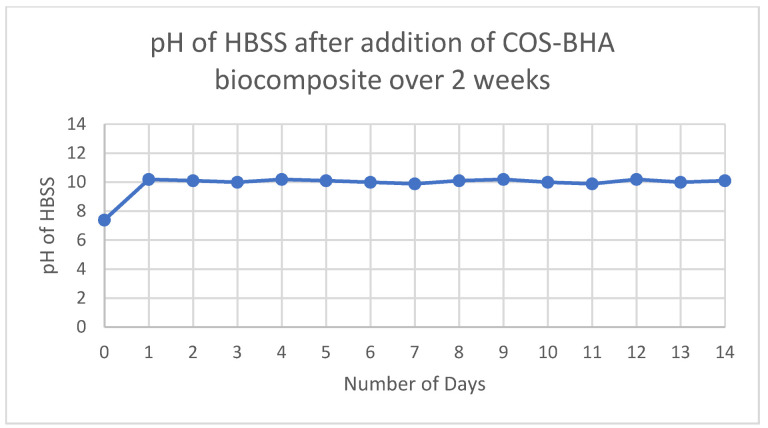
pH of HBSS over 2 weeks following the addition of the experimental pulp-capping agent.

Unset Sample 4 showed a pH of 10, as indicated by the pH indicator strip. When placed into the HBSS, the pH of the solution gradually rose and yielded a reading of 10.2 after 24 h. This pH reading stayed consistent over the next two weeks of monitoring, with a reading of 10.1 at the end of 14 days (Figure 3).

**Figure 4:**
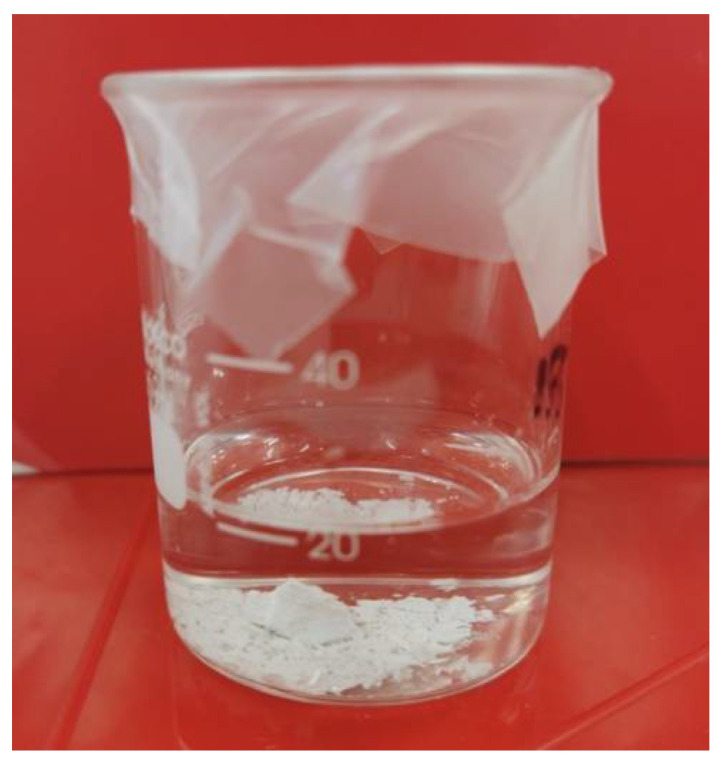
An amount of 1.5 g of set experimental pulp-capping material Sample 4 in 20 mL of Hank’s Balanced salt solution.

When placed into HBSS, the exterior surface of the set Sample 4 slowly began to break down and the structure crumbled over a 2 min period. Without agitation, the core structure remained largely intact over the 2 week period, as illustrated in Figure 4. BHA and ZrO_2_ did not solubilise in the HBSS.

### 3.6. Biocomposite Setting Time 

It took on average 1 h and 10 min for Sample 4 to set to a point where the Vicat apparatus no longer caused an indentation on its surface. Anecdotally, the material seemed to reach its full strength and feel dry to the touch after 2 h.

### 3.7. Biocomposite Radiopacity 

**Figure 5:**
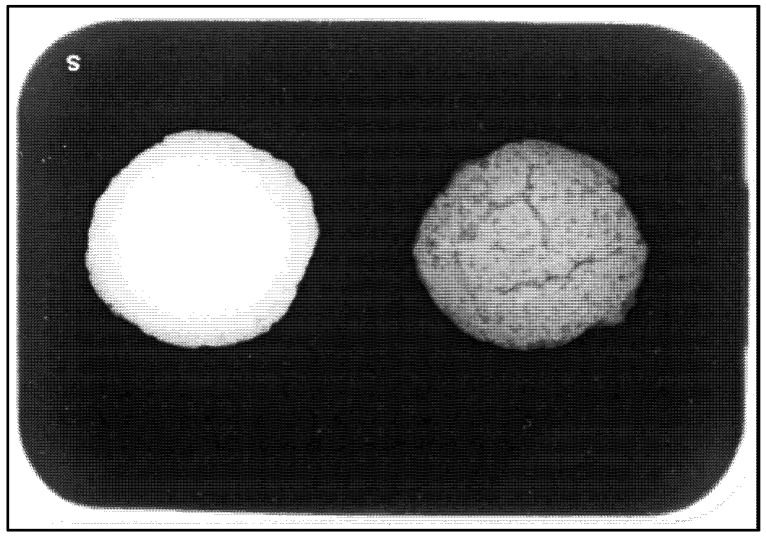
Radiograph of (**left**) Sample 4 experimental pulp-capping agent and (**right**) MTA.

The experimental pulp-capping agent demonstrated greater radiopacity than that of MTA (Figure 5).

## 4. Discussion

The current study aimed to develop and investigate the feasibility of a hybrid composite material using COS and BHA for vital pulp therapy. Both COS and BHA have biological properties that render them promising materials as pulp-capping agents. Additionally, both materials can be sourced from waste products, meaning that any pulp-capping agent derived from the two could provide an economical alternative to current pulp-capping materials on the market. ZrO_2_ was chosen as the radiopacifier over bismuth oxide commonly seen in MTA because bismuth oxide reportedly exhibits toxicity towards human dental pulp cells [17]. In contrast, ZrO_2_ is bioinert, biocompatible, and widely used in the repair of dental and skeletal tissues [17]. A fixed ratio of 35% wt ZrO_2_ was used for all tested samples, as prior research indicated that the addition of ZrO_2_ in concentrations above 20% wt results in radiopacity equal to or exceeding that of MTA [13]. Of the five investigated samples, the proportion of COS solution in Samples 1 and 2 was too high; as a result, the BHA and ZrO_2_ powders demonstrated sedimentation. On the other hand, the proportions outlined for Sample 5 resulted in a mixture that was too dry, and the BHA and ZrO_2_ powders were unable to be mixed homogeneously. For this reason, Samples 1, 2, and 5 were deemed to be unsuitable for pulp capping. Samples 3 and 4 were both promising, yielding a homogenous and injectable paste. However, Sample 3, with its higher proportion of COS solution, took longer to set and resulted in a more friable set product compared to Sample 4. For this reason, Sample 4 was selected for the characterisation of its physicochemical properties.

The FTIR analysis of the COS, BHA, and ZrO_2_ powders confirmed that the raw materials match the known FTIR spectra from previous studies [14,15,16]. The COS spectrum showed an absorption band at 3275.89 cm^−^^1^ representing NH and –OH stretching vibrations. A weak band at 2869.91 cm^−^^1^ was attributed to –CH stretching, and 1588.33 cm^−^^1^ was assigned to either the amide I or II absorption band. The absorption band at 1374.31 cm^−^^1^ coincided with the deformation vibrations of –CH_2_. Bands 1150. and 1030.98 cm^−^^1^ were consistent with –CO stretching in secondary alcohol and the asymmetric stretching of the –C–O–C in the skeletal linkage of COS, respectively [14]. BHA demonstrates phosphate absorption bands at 1087.74–1026.38, 962.07, and 599.34–563.12 cm^−^^1^. Bands at 3571 cm^−^^1^ and 628.53 cm^−^^1^ coincided with functional groups of OH– in stretching and liberational mode [15]. The FTIR spectra of BHA demonstrated similar absorption peaks to synthetic hydroxyapatite, indicating that the bovine bone was successfully processed to remove fat and protein components. The spectrum for ZrO_2_ demonstrates absorption bands at 726.34 and 671.31 cm^−^^1^, consistent with the stretching vibration of the Zr–O bond [16]. FTIR analysis for the selected hybrid biocomposite produced a spectrum demonstrating peaks overlapping the spectra of COS, BHA, and ZrO_2_, representing the formation of a composite of the combined raw materials.

ICP-MS analysis of the experimental pulp-capping material revealed the main inorganic element constituents of the sample to be zirconium, calcium, phosphate, sodium, and magnesium. Zirconium can be attributed to the ZrO_2_ powder (Table 2). While the latter elements were attributed to the BHA powder, with calcium and phosphate being predominant elements present in hydroxyapatite, sodium, and magnesium trace elements commonly found in bovine derived hydroxyapatite [10]. Similarly, EDX spectra also confirmed zirconium, calcium, phosphate, sodium, and magnesium to be the predominant inorganic elements along with the organic element carbon. Carbon was attributed to the presence of COS. ICP-MS analysis for the presence of toxic heavy metals reveals that arsenic, lead, and cadmium levels were <5, <0.2, and <2 mg/kg, respectively. The levels of lead and cadmium detected within the sample were lower than the allowed levels suggested under ISO 9917-1 guidelines and the ASTM standard (F1185-03). The level of detected arsenic exceeded the allowable level of <2 mg/kg, as outlined by the ISO 9917-1 guidelines. However, the ISO document outlines the allowable levels of leachable arsenic and uses a method whereby the set material is crushed and soaked in 20% HCl solution for 16 h to leach the arsenic and lead [18]. In contrast, the experimental pulp-capping material assessed in this study was digested using concentrated HNO_3_, Hf, and HCl. Therefore, the levels of detected arsenic represent the total arsenic levels present and are unlikely to be representative of the leachable arsenic content, which is likely lower. Furthermore, the relevance of ISO 9917-1 was questioned, since crushing the material increases the surface area beyond what would occur clinically [18]. Further research is necessary to determine the level of leachable arsenic within the sample as outlined by ISO 9917-1.

In terms of the physical properties of the experimental pulp-capping agent, a high pH of 10 is beneficial for pulp capping. Existing pulp-capping materials in calcium hydroxide and MTA both have a pH of 12, which confers antibacterial properties and helps in inducing the deposition of reparative dentine [4]. A limitation of this study is that antimicrobial activity was not investigated, which should be a future direction. The experimental pulp-capping material also demonstrated good radiopacity by incorporating the ZrO_2_ powder. The experimental pulp-capping material was clearly more radiopaque than MTA in this study (Figure 5), suggesting that the radiopacity of the material is excellent for easy clinical identification.

However, the current iteration of the experimental pulp-capping material also has drawbacks. The average setting time of 1 h and 10 min, while shorter than the setting time of MTA at 2 h and 45 min, would still require the clinician to either deliver the treatment in two stages or place a liner prior to the permanent restoration. Second, the dissolution of the set material in HBSS indicates that the material is likely to separate into its individual components over time in the presence of fluid. This is less relevant for the placement of the material, as CPT is conducted under dental dam isolation, which reduces the potential for washing out the material during its placement [13]. However, the breakdown of the material over time within the pulp chamber could lead to microleaks in the absence of a good orifice barrier and permanent restoration [19]. Therefore, future studies may look at incorporating a crosslinking agent into the formulation to improve the structural integrity of the experimental pulp-capping material.

## 5. Conclusions

This study demonstrated the development of a COS-BHA hybrid biocomposite as a potential pulp-capping agent for conservative pulp therapy. The COS–BHA hybrid biocomposite can be produced from waste products, providing a cheaper endodontic material compared to conventional pulp-capping agents such as MTA. The characterisation of the material’s physical properties yielded promising results. The material exhibited a high pH of 10, greater radiopacity, and shorter setting time than MTA did. Future studies will look to performing in vitro and in vivo tests on the COS–BHA hybrid biocomposite to assess its in vitro biocompatibility, biological properties, and clinical feasibility. The incorporation of crosslinking agents may also be investigated to reduce the solubility of the material.

## Figures and Tables

**Table 1:** Proportions of COS, BHA, and ZrO_2_ via percentage by weight (wt) for each investigated sample.

Sample	COS Sol (% by wt)	COS Sol (g)	BHA (% by wt)	BHA (g)	ZrO_2_ (% by wt)	ZrO_2_ (g)
1	55	5	10	0.91	35	3.18
2	45	5	20	2.22	35	3.89
3	35	5	30	4.29	35	5.00
4	25	5	40	8.00	35	7.00
5	15	5	50	16.67	35	11.67

**Table 2:** Elemental composition of experimental pulp-capping material determined by ICP-MS following digesting in concentrated HNO_3_, HF, and HCI.

Element (mg/kg)	Experimental Pulp-Capping Material
Zr	68,100
Ca	55,300
P	14,600
Na	1320
Mg	914
K	<900
Al	<200
B	<200
Zn	<90
Fe	<50
Sr	43
Ba	19

Elements detected at levels below 5 mg/kg are not presented.

**Table 3:** Composition of the experimental pulp-capping agent determined by SEM-EDX.

Element	Weight (%)
C	13.47
O	31.02
Na	1.17
Mg	1.28
P	2.66
Ca	6.01
Zr	43.72
Hf	0.66
Total	100

## Data Availability

The data presented in this study are available on request from the corresponding author.
